# Cannabidiol attenuates insular dysfunction during motivational salience processing in subjects at clinical high risk for psychosis

**DOI:** 10.1038/s41398-019-0534-2

**Published:** 2019-08-22

**Authors:** Robin Wilson, Matthijs G. Bossong, Elizabeth Appiah-Kusi, Natalia Petros, Michael Brammer, Jesus Perez, Paul Allen, Philip McGuire, Sagnik Bhattacharyya

**Affiliations:** 1 Department of Psychosis Studies, Institute of Psychiatry, Psychology and Neuroscience, King’s College London, London, UK; 2 Department of Psychiatry, Brain Center Rudolf Magnus, University Medical Center Utrecht, Utrecht, Netherlands; 3 Centre for Neuroimaging Sciences, Department of Neuroimaging, Institute of Psychiatry, Psychology, and Neuroscience, King’s College London, London, UK; 4 CAMEO Early Intervention Service, Cambridgeshire and Peterborough NHS Foundation Trust, Cambridge, UK; 5 Cognition, Neuroscience and Neuroimaging (CNNI) Laboratory, Department of Psychology, University of Roehampton, London, UK

**Keywords:** Pharmacodynamics, Schizophrenia

## Abstract

Accumulating evidence points towards the antipsychotic potential of cannabidiol. However, the neurocognitive mechanisms underlying the antipsychotic effect of cannabidiol remain unclear. We investigated this in a double-blind, placebo-controlled, parallel-arm study. We investigated 33 antipsychotic-naïve subjects at clinical high risk for psychosis (CHR) randomised to 600 mg oral cannabidiol or placebo and compared them with 19 healthy controls. We used the monetary incentive delay task while participants underwent fMRI to study reward processing, known to be abnormal in psychosis. Reward and loss anticipation phases were combined to examine a motivational salience condition and compared with neutral condition. We observed abnormal activation in the left insula/parietal operculum in CHR participants given placebo compared to healthy controls associated with premature action initiation. Insular activation correlated with both positive psychotic symptoms and salience perception, as indexed by difference in reaction time between salient and neutral stimuli conditions. CBD attenuated the increased activation in the left insula/parietal operculum and was associated with overall slowing of reaction time, suggesting a possible mechanism for its putative antipsychotic effect by normalising motivational salience and moderating motor response.

## Introduction

The aberrant salience hypothesis of psychosis^1^ postulates that hyperdopaminergia in the mesostriatal pathway leads to aberrant assignment of salience to everyday experiences and stimuli, which in turn result in psychotic symptoms. Elevated presynaptic dopamine function in the striatum is established in psychotic disorders^2^ and in subjects at clinical high risk for psychosis (CHR)^3,4^, and an understanding of the relationship between dopamine, aberrant salience and psychotic symptoms, particularly delusions, is emerging^5^. It has been suggested that mesostriatal dopaminergic overactivity may be driven by glutamatergic dysfunction in the medial temporal lobe (MTL)^6^, and both increased hippocampal blood flow^7^ and metabolism^8^ have been reported in CHR subjects and established psychosis.

Dopamine signalling is fundamental to reward processing^9^ which is dysfunctional in psychosis^10^. Reward processing includes the attribution of ‘motivational salience’, whereby the anticipation of a rewarding stimulus or incentive prepares an individual for ‘approach behaviour’ towards eventual consumption. Neuroimaging studies have demonstrated abnormal brain activity during cognitive tasks capturing ‘motivational salience’ in CHR and psychosis. Compared to healthy controls, CHR subjects have been found to have hypoactivation in the ventral striatum (VS) and midbrain^11^ and right inferior parietal lobule^12^, with VS activity to ‘aberrant’ or non-salient stimuli correlating with severity of positive psychotic symptoms^13^. Others have shown increased activation in the posterior cingulate cortex (PCC), middle and superior frontal gyri (MFG, SFG)^14^ and ventral pallidum and midbrain^15^. In the VS, activity while processing both non-salient^13^ and salient stimuli^15^ have been correlated with positive psychotic symptoms, as has activity in the right anterior insula during salient stimuli^15^. In established psychosis, meta-analysis suggests hypoactivation of the VS^16^, and individual studies have reported reduced activation in the cingulate and ventral tegmentum in unmedicated patients^12,17^, and in the right insula in medicated patients^12^ while processing motivational salience.

While the aberrant salience hypothesis of psychosis generally focuses on midbrain and striatal function, emerging evidence points towards a key role for other brain regions. In particular, the ‘salience network’ (SN), anchored in the anterior cingulate (ACC) and insular cortex (IC), may play a role in selecting relevant internal and externally generated signals for higher order processing^18,19^. Altered volume, activation and dysconnectivity of components of the SN have been observed both in established psychotic disorders^12,20–22^ and in CHR^23–26^ prior to the onset of psychosis, with evidence of association between symptoms and both the extent of volume loss^27^ and altered activation^12,28,29^ of the insula. This has led to the hypothesis that psychotic symptoms arise as a result of insular dysfunction within the salience network^30^.

There is evidence from healthy volunteer studies that cannabidiol (CBD), a non-psychoactive substance in cannabis, opposes the psychotomimetic effects of Δ9-tetrahydrocannabinol (Δ9-THC)^31,32^, its main psychoactive ingredient. This is complemented by evidence of efficacy as an antipsychotic in some^33,34^, though not all^35^, clinical trials. We have recently shown that CBD may normalise MTL, midbrain and striatal dysfunction in CHR patients^36^, but the precise neurocognitive mechanism of any antipsychotic effect remains unclear. Whether CBD modulates aberrant motivational salience, and whether this is linked to any antipsychotic effect remains untested.

Therefore, in this study we investigated whether there is a pattern of abnormal activation in CHR compared to healthy controls during the processing of motivationally salient stimuli, and whether a single dose of CBD attenuates this abnormal function in CHR. We selected CHR subjects, because they are antipsychotic-naïve and at risk of developing psychosis^37^, thus avoiding confounding effects of dopamine antagonism. Furthermore, they are more stable than people with established psychosis and can better tolerate the demands of complex neuroimaging investigations.

We employed the monetary incentive delay task (MIDT), a reward processing task adapted for fMRI^38^. The MIDT allows reward processing to be parsed into at least two distinct components: ‘anticipation’ and ‘feedback’. We focused on the anticipation condition, as VS activity in this condition has been linked to dopamine release^39^, and the SN is robustly activated in both anticipation of reward and loss^40^. Hence, we did not limit anticipation to one specific valence (e.g. reward or loss), but combined all motivationally salient conditions, as previously reported^17,41^. Existing research in CHR using the MIDT has found abnormal activation in the PCC, MFG and SFG in reward anticipation^15^, though abnormal striatal activity hasn’t been detected^15,42^.

Our primary hypothesis was that CHR participants would display altered activation in the core SN (IC and ACC) relative to healthy controls, and a single dose of CBD would have an opposite effect in these regions. Our secondary hypothesis was that CHR participants would display altered activation in the midbrain, striatum and hippocampus, and again CBD would have an opposite effect in these regions.

## Method

### Participants

Thirty-three CHR participants aged 18–35 years were recruited from early intervention services in the UK. Exclusion criteria included history of psychotic or manic episode, current DSM IV diagnosis of substance dependence (except cannabis), neurological disorder or severe intercurrent illness, unwillingness to use barrier contraception, pregnancy, and any contraindication to MRI. All participants gave written, informed consent. Participants were required to abstain from cannabis for 96 h, other recreational substances for 2 weeks, alcohol for 24 h and caffeine and nicotine for 6 h before attending. Urine samples were collected prior to drug administration to monitor for substance use and to exclude pregnancy. Nineteen healthy control (HC) participants matched for age (within 3 years), sex and ethnicity were recruited by local advertisement. All participants gave written informed consent prior to commencing the trial. The study was approved by the National Research Ethics Service Committee of London—Camberwell St Giles.

### Study design and measures

This study was a randomised placebo-controlled double-blind, parallel-arm fMRI investigation of the acute effect of 600 mg oral CBD on the anticipation phase of the MIDT in subjects deemed at clinical high-risk of psychosis. Randomisation and blinding were carried out at the Maudsley Hospital Pharmacy. Psychopathology was assessed by a trained interviewer using the Comprehensive Assessment of At-Risk Mental States interview (CAARMS)^37^ prior to drug administration. Plasma CBD levels were sampled 120 and 300 min after drug administration. MRI scanning took place 180 minutes after drug intake. Participants were monitored for any adverse reactions. The study took place at the Clinical Research Facility, King’s College Hospital and the Centre for Neuroimaging Sciences, Department of Neuroimaging, Institute of Psychiatry, Psychology and Neuroscience.

### Monetary incentive delay task

Participants underwent two runs of the MIDT each consisting of 48 individual trials. Four conditions were used, induced by learned visual cues: neutral (£0), win small (£0.20), win large (£2.00) and lose (£2). Participants underwent standardised training prior to entering the scanner. There were 12 trials for each condition randomised into 48 trials per run, with two consecutive runs lasting 8 min each. Participants began each run with a baseline figure of £10.00 and received payment at the end of the same study day for the cumulative total won in both runs.

The cue was presented for 250 ms and the feedback for 1450 ms (see Supplementary Fig 1). Target presentation time varied for each run by ±10ms from an initial 250 ms and ranging between 150 and 300 ms to assure ~66% success for each participant. A successful hit depended on the participant responding by pressing the button during target presentation. A response prior to 100 ms after target onset was considered an unsuccessful ‘false-start’. Scanning of anticipation occurred during the interval between cue and target which varied from 3700 to 4500 ms in duration. The inter-trial interval was 10 s for all trials.

### Drug intervention

CHR participants were randomised to receive either oral 600 mg CBD (CHR-CBD; CBD obtained from THC Pharm, Germany) or placebo (CHR-PLB) prepared in identical capsules following a standard light breakfast. Participants were administered the capsule at ~11 a.m., 180 min before the start of scanning.

### Scanning parameters

Participants underwent structural and functional MRI in a single session. Images were acquired using a General Electric Signa HDx 3.0 T MRI scanner. Structural images were acquired using a whole-brain sagittal T1-weighted scan based on Alzheimer’s Disease Neuroimaging Initiative parameters (TE = 2.85 ms, TR = 6.98 ms, inversion time = 400 ms, flip angle = 11^0^, voxel size 1.0 × 1.0 × 1.2 mm). 480 T2*-weighted images were acquired in two 8-min runs (TE = 30 ms, TR = 2.0 s, flip angle = 75°, 39 × 3 mm thick axial planes, 3.3 mm inter-slice gap, in-plane voxel size 3.75 × 3.75 mm).

### Analysis

#### Imaging

fMRI data were preprocessed using SPM8 (Wellcome Trust Centre for Neuroimaging) by realignment of functional images, co-registration with the structural scan, spatial normalization into standard MNI space and smoothing by a Gaussian filter (FWHM = 8 mm). Using general linear model regression with factors time-locked to task events and convolved with a canonical hemodynamic response function, the regression coefficient (*b*-value) for each voxel was determined. There were 12 regressors in the task design: four modelling conditions of anticipation (anticipation win large £2, anticipation win small 20p, anticipation lose £2 and anticipation neutral), seven modelling feedback conditions (neutral feedback following anticipation neutral and successful or unsuccessful response feedback for the remaining six anticipation conditions) and one regressor modelling response activity for all four anticipation conditions. Within-group maps were created for salience condition by combining anticipation of all win and loss conditions and contrasting with neutral anticipation. Between-group contrasts were created comparing HC with CHR-PLB (HC-vs-CHR-PLB) and CHR-PLB with CHR-CBD (CHR-PLB-vs-CHR-CBD).

Two region-of-interest (ROI) analyses were performed using masks created for the SN and combined hippocampus-midbrain–striatum (HMS). The SN mask was created using the Pick Atlas in SPM8 by selecting human bilateral ACC and insulae. The HMS mask was defined by a previous study of CHR^7^ and consisted of bilateral medial hippocampi, subicula, caudate, putamen, pallidum and midbrain. Exploratory whole-brain analysis was also conducted.

To test the hypothesis that activation in CHR-CBD would be intermediate between that of HC and CHR-PLB, we examined whether a linear relationship in brain activation (CHR-PLB > CHR-CBD > HC) existed within the ROI’s and at whole-brain level by three-way ANOVA. We applied a family-wise error corrected (FWE) *p* < 0.05 threshold, corrected for volume for all analyses.

#### Behavioural performance

Behavioural performance was analysed for the two between-group contrasts of interest (HC-vs-CHR-PLB and CHR-PLB-vs-CHR-CBD), including five components: mean monetary reward (£GBP), accuracy (percentage response on target), reaction time (ms), false-starts (premature action initiation) and any trial responses (attention, percentage).

Pairwise independent *t*-testing was applied for mean monetary reward, pairwise ANOVA for mean reaction time, and pairwise binary logistic regression for accuracy, false-starts, delayed reaction and any trial response.

We tested for correlation between activation and behavioural performance (RT) and psychotic symptoms using the mean *b*-value for ANOVA-derived clusters.

## Results

There was no significant difference between HC (*n* = 19), CHR-PLB (*n* = 17) and CHR-CBD (*n* = 16) in age, gender, ethnicity, country of birth or handedness (see Table 1). There were no significant differences between CHR-PLB and CHR-CBD in either positive or negative symptom subscale of the CAARMS or in terms of current tobacco smoking and cannabis use. HC participants were selected to have minimal drug use history. In the CHR-CBD group, mean plasma CBD levels were 126.4 nM (sd 221.8) before and 823.0 nM (sd 881.5) after the fMRI scan.

**Table 1:** Sample characteristics

	HC (*n* = 19)	CHR-CBD (*n* = 16)	CHR-PLB (*n* = 17)	Pairwise analysis	
				HC-vs-CHR-PLB	CHR-PLB-vs-CHR-CBD
Age/yr (sd)	23.9 (4.15)	22.7 (5.08)	24.1 (4.48)	*p* = 0.91^a^	*p* = 0.42^a^
Ethnicity %					
White	57.9	62.5	41.2	*p* = 0.59	p = 0.43^b^
Black White	26.3	12.5	29.4		
Asian	0	0	5.9		
Mixed	15.8	25	23.5		
UK born %	57.9	68.8	82.4	*p* = 0.26^b^	*p* = 0.51^b^
Years education (sd)	17.0 (1.58)	14.5 (3.06)	11.9 (3.44)	***P*** **<** **0.01**^**a**^	*p* = 0.09^a^
Gender % (male)	57.9	62.5	41.2	*p* = 0.32^b^	*p* = 0.22^b^
UDS % (positive)	0	63	47	Not compared^c^	*p* = 0.45^b^
THC	0	13	29		
Morphine	0	6	0		
Benzodiazepine	0	0	6		
Phencyclidine	0	0	6		
Missing	0	19	12		
Current smoker % (yes)	10.5	31.3	56.3	Not compared^c^	*p* = 0.14^b^
Current cannabis use %	0	43.8	41.2	Not compared^c^	*p* = 0.88^b^
Handedness % (right)	94.7	87.5	100	*p* = 0.38^b^	*p* = 0.16^b^
*CAARMS score (sd)*					
Positive symptoms	NA	40.19 (20.79)	42.94 (29.46)	NA	*p* = 0.75^a^
Negative symptoms	NA	23.25 (16.49)	28.41 (10.17)	NA	*p* = 0.43^a^

*HC* healthy control group, *CHR-CBD* clinical-high risk cannabidiol group, *CHR-PLB* clinical-high risk placebo group, *CAARMS* comprehensive assessment of at-risk mental state

^a^Independent *t*-test

^b^Pearson chi-squared test

^c^HC were selected to have minimal drug use and hence were not compared with CHR participants on these parameters

Bold values indicates statistical significance in respective tests

### Behavioural performance

Mean monetary reward: at the end of the 96 trials (2 runs of 48), the HC group appeared to win a higher cumulative total of money, though this was non-significant in pairwise analysis (Table 2).

**Table 2:** Behavioural performance

	HC	CHR-CBD	CHR-PLB	Pairwise analysis		
				HC-vs-CHR-PLB	HC-vs-CHR-PLB	
Mean monetary reward £GBP (SD)	41.00 (6.63)	37.48 (12.14)	36.13 (11.97)	*p* = 0.150^a^	*p* = 0.751^a^	
*Accuracy (successful hits on target) %*						
Overall	63.5	60.4	59.3	Group exp(B) = 1.147 (CI 0.981–1.341), *p* = 0.085^b^ Condition exp(B) = 0.639 (CI 0.546–0.747), ***p*** **<** **0.001**^b^ Condition ✲ group exp(B) = 0.841 (CI 0.615–1.149), *p* = 0.276^b^	Group exp(B) = 0.985 (CI 0.837–1.159), *p* = 0.855^b^ Condition exp(B) = 0.616 (CI 0.0.523–0.724), ***p*** **<** **0.001**^b^ Condition ✲ group exp(B) = 0.780 (CI 0.564–1.080), *p* = 0.134^b^	
Neutral	53.9	49.2	52.7			
Salience	66.7	64.1	61.5			
*Mean reaction time/ms* *>* *100* *ms (SD)*						
Overall	243.13 (44.97)	251.31 (52.19)	245.70 (46.82)	Group F(1,3275) = 0.207, *p* = 0.649^c^ Condition F(1,3275) = 36.60, ***p*** **<** **0.001**^**3**^ Condition ✲ group F(1,3275) = 2.974, *p* = 0.085^c^	Group F(1,3151) = 16.67, ***p*** **<** **0.001**^**c**^ Condition F(1,3151) = 51.500, ***p*** **<** **0.001**^**c**^ Condition ✲ group F(1,3151) = 0.000, *p* = 0.992^c^	
Neutral	254.32 (49.36)	262.68 (58.60)	251.93 (52.02)			
Salience	239.69 (42.97)	248.00 (49.72)	243.79 (44.96)			
*False starts %*						
Overall	0.9	1.8	3.2	Group exp(B) = 4.630, (CI 2.054–10.437), ***p*** **<** **0.001**^**b**^ Condition exp(B) = 1.446 (CI 0.642–3.260), *p* = 0.374^b^ Condition ✲ group exp(B) = 2.224 (CI 0.438–11.303), *p* = 0.335^b^	Group exp(B) = 1.678 (CI 0.970–2.902), *p* = 0.064^b^ Condition exp(B) = 0.875 (CI 0.506–1.513), *p* = 0.632^b^ Condition ✲ group exp(B) = 0.814 (CI 0.272–2.435), *p* = 0.712^b^	
Neutral	0.5	2.2	3.2			
Salience	1.0	1.7	3.1			
*Delayed response %*						
Overall	33.2	32.8	35.0	Group exp(B) = 0.995, (CI 0.843–1.175), *p* = 0.953^b^ Condition exp(B) = 0.733 (CI 0.621–0.866, ***p*** **<** **0.001**^b^ Condition ✲ group exp(B) = 0.816 (CI 0.585–1.139), *p* = 0.232^b^		Group exp(B) = 1.035 (CI 0.867–1.235), *p* = 0.704^b^ Condition exp(B) = 0.747 (CI 0.626–0.892), ***p*** **=** **0.001**^b^ Condition ✲ group exp(B) = 0.848 (CI 0.595–1.208), *p* = 0.362^b^
Neutral	40.3	39.6	38.8			
Salience	31.0	30.8	33.9			
*Trial response %*						
Overall	96.8	93.0	96.6	Group exp(B) = 0.939, (CI 0.624–1.415), *p* = 0.765^b^ Condition exp(B) = 7.667 (CI 5.092–11.546), ***p*** **<** **0.001**^b^ Condition ✲ group exp(B) = 0.810 (CI 0.488–2.507), *p* = 0.810^b^		Group exp(B) = 2.330 (CI 1.635–3.321), ***p*** **<** **0.001**^**b**^ Condition exp(B) = 5.754 (CI 4.037–8.202), ***p*** **<** **0.001**^b^ Condition ✲ group exp(B) = 0.623 (CI 0.307–1.265), *p* = 0.190^b^
Neutral	90.8	84.1	90.7			
Salience	98.8	96.0	98.6			

*HC* healthy control group, *CHR-CBD* clinical-high risk cannabidiol group, *CHR-PLB* clinical-high risk placebo group

^a^Independent *t*-test

^b^Pairwise binary logistic regression

^c^Analysis of variance

Bold values indicates that the result of the statistical test was significant

Accuracy: There was a significant likelihood of increased accuracy in the salience condition compared to neutral in both HC-vs-CHR-PLB (*p* < 0.001) and CHR-PLB-vs-CHR-CBD (*p* < 0.001). There was a trend toward impaired accuracy in CHR-PLB compared to HC across all stimuli conditions (*p* = 0.085), but there was no interaction between salience and group. There was no significant difference between CHR-PLB and CHR-CBD or group by condition interaction.

**Fig. 1:**
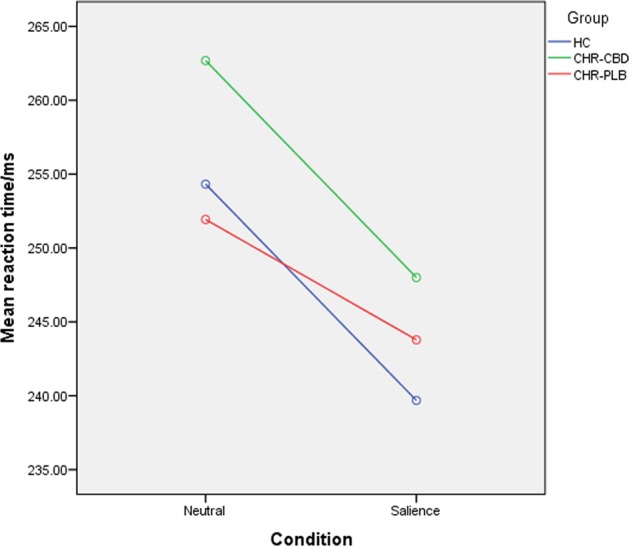
Mean reaction time by condition by group

Reaction time (see Fig. 1): RT shortened significantly in the salience condition compared to the neutral stimuli condition for both HC-vs-CHR-PLB (*p* < 0.001) and CHR-PLB-vs-CHR-CBD (*p* < 0.001). Regarding HC-vs-CHR-PLB, there was a trend-level interaction between group and condition (*p* = 0.085) such that the acceleration of response (as indexed by shorter RT) while viewing salient stimuli compared to neutral stimuli was greater in HC than in CHR-PLB. RT was significantly slower overall in CHR-CBD than CHR-PLB (*p* < 0.001).

False-starts (premature action initiation): CHR-PLB were significantly more likely to produce false-starts than HC (*p* < 0.001) and CHR-CBD at trend-level (*p* = 0.064). There were no significant effects of condition or group by condition interaction in either HC-vs-CHR-PLB or CHR-PLB-vs-CHR-CBD.

Trial response: in both pairwise analyses, subjects were more likely to respond in the salience condition (HC-vs-CHR-PLB *p* < 0.001; CHR-PLB-vs-CHR-CBD *p* < 0.001). There was no difference between CHR-PLB and HC, but CHR-CBD was significantly less likely to respond than CHR-PLB (*p* < 0.001). There was no group by condition interaction in either HC-vs-CHR-PLB or CHR-PLB-vs-CHR-CBD.

### Imaging

A single participant in the CBD group was excluded from imaging analysis because of inattention to all neutral trials with a subsequent lack of corresponding contrasts, such that the imaging sample sizes were 19 (HC), 15 (CHR-CBD) and 17 (CHR-PLB).

### Task network (HC only)

In HC, salience condition was associated with activation in both SN and HMS masks and across the whole brain (see Supplementary Table 1).

### HC-vs-CHR-PLB

**Fig. 2:**
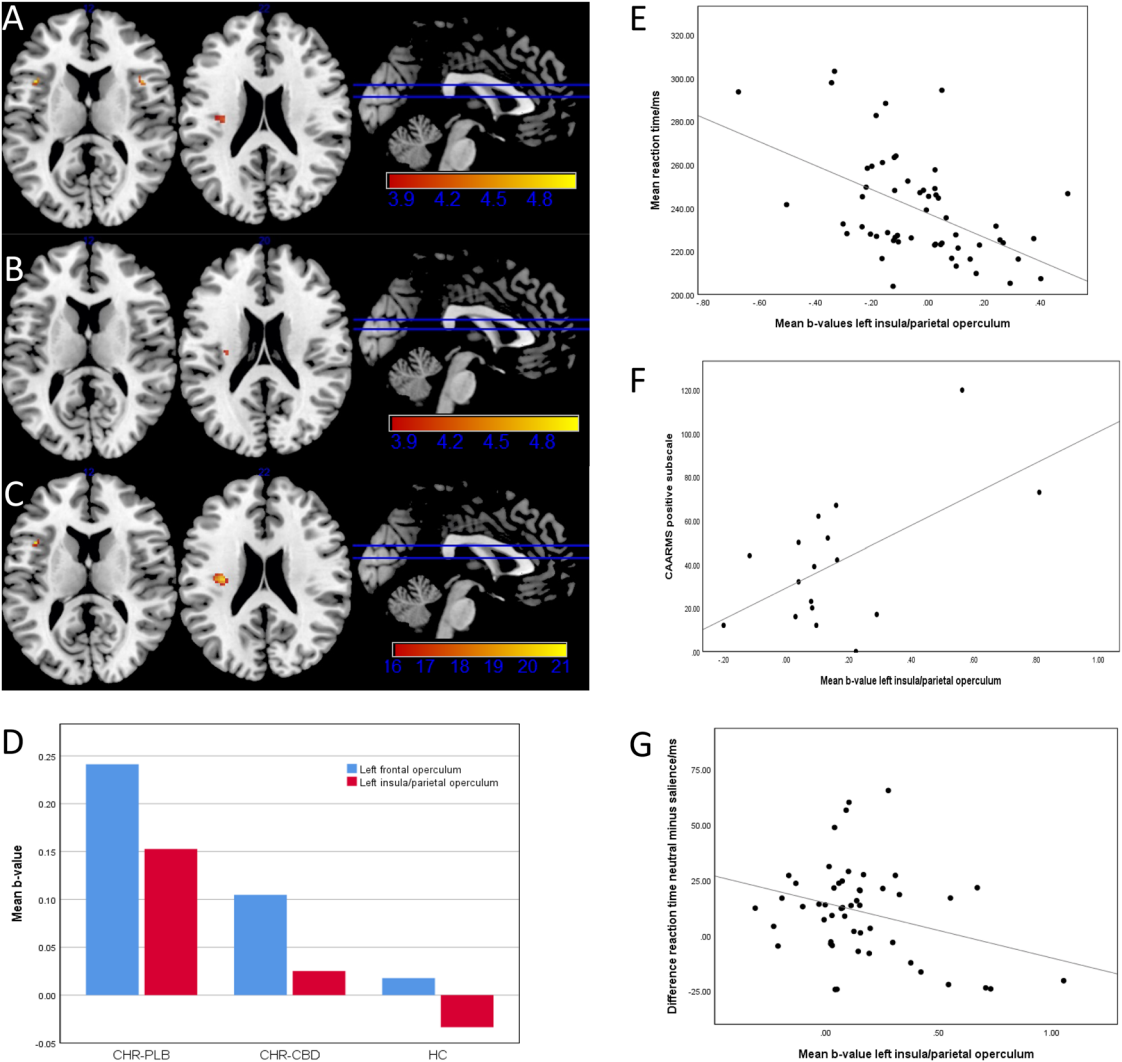
Salience network region-of-interest analysis of salience>neutral contrast (small-volume corrected, *p* < 0.05 FWE-corrected at voxel level, *k* ≥ 3 voxels). **a** Pairwise comparison CHR-PLB>HC with clusters in bilateral frontal operculae and left insula/parietal operculum. **b** Pairwise comparison CHR-PLB>CHR-CBD with cluster in left insula/claustrum. **c** Three-way ANOVA CHR-PLB>CHR-CBD>HC with clusters in left frontal operculum and left insula/parietal operculum. **d** Mean *b*-value parameter estimates extracted from the two clusters generated by ANOVA for each group (CHR-PLB, CHR-CBD, and HC) showing increased activation in CHR-PLB relative to HC with CHR-CBD intermediate in the left frontal operculum and left insula/parietal operculum. **e** Negative correlation between mean b-value from ANOVA-derived cluster of left insula/parietal operculum and mean reaction time for salience condition in HC. **f** Positive correlation between mean b-value from ANOVA-derived cluster of left insula/parietal operculum and CAARMS positive subscale in CHR-PLB. **g** Negative correlation between mean b-value from ANOVA-derived cluster of left insula/parietal operculum and difference in mean reaction time between neutral and salience condition in CHR-PLB

Within the SN (Table 3, Fig. 2), the bilateral frontal operculae (FO; left: *k* = 12 voxels, *T* = 4.77, *p* = 0.002; right: *k* = 18 voxels, *T* = 4.47, *p* = 0.006) and the left insula converging with left parietal operculum (PO; *k* = 13 voxels, *T* = 4.11, *p* = 0.019) were significantly more active in CHR-PLB compared to HC during salient compared to neutral condition. No areas met significance threshold for HMS. At whole-brain level (Supplementary Table 2, Fig. 2), the following regions were significantly more active in CHR-PLB: the left SFG medial part (*k* = 141 voxels, *T* = 6.55, *p* < 0.001), a cluster spanning the left inferior frontal gyrus opercular part and left FO (*T* = 5.47, *p* = 0.002; *T* = 5.26, *p* = 0.004), and the left superior temporal gyrus (*k* = 13 voxels, *T* = 5.06, *p* = 0.009).

**Table 3:** Salience network analysis for salience-vs-neutral contrast

Region	Peak coordinate (MNI)			Cluster size	*p* value
	*x*	*y*	*z*		
Pairwise comparison CHR-PLB ***>*** HC					
Left frontal operculum	−42	14	12	12	0.002
Right frontal operculum	42	14	12	18	0.006
Left insula/parietal operculum	−32	−16	22	13	0.019
Pairwise comparison CHR-PLB ***>*** CHR-CBD					
Left insula/claustrum	−30	−16	20	3	0.035
Three-way ANOVA CHR-PLB **>** CHR-CBD **>** HC					
Left frontal operculum	−42	14	22	6	0.007
Left insula/parietal operculum	−32	−16	22	26	0.009

Small volume corrected, family wise error-corrected *p* < 0.05, *k* ≥ 3 voxels

*HC* healthy control group, *CHR-CBD* clinical-high risk cannabidiol group, *CHR-PLB* clinical-high risk placebo group

### CHR-PLB-vs-CHR-CBD

Within the SN (Table 3, Fig. 2), the left insula/claustrum (*k* = 3 voxels, *T* = 3.98, *p* = 0.035) was more active in CHR-PLB compared to CHR-CBD during salient relative to neutral condition. No areas met significance threshold for HMS. At whole-brain level (Supplementary Table 2, Fig. 2), the right SFG lateral part was more active in CHR-PLB (*k* = 3 voxels, *T* = 4.99, *p* = 0.025), and the right cerebellum posterior lobe was more active in CHR-CBD (*k* = 6 voxels, *T* = 5.03, *p* = 0.022).

### Between-group linear analysis

ANOVA of the SN (CHR-PLB > CHR-CBD > HC; Table 3, Fig. 2) during salient relative neutral condition generated two significant peaks. The largest was located in the left insula/PO (*k* = 26 voxels, *F* = 20.13, *p* = 0.009) with the exact same peak coordinate reported in HC-vs-CHR-PLB (−32, −16, 22). The second was located in the left FO (*k* = 6 voxels, *F* = 20.65, *p* = 0.007). Mean b-values for each group confirmed increased activation in CHR-PLB compared to HC, with CHR-CBD intermediate. No areas met significance threshold for HMS. Exploratory whole-brain ANOVA (CHR-PLB > CHR-CBD > HC; Supplementary Table 2, Fig. 2), generated a significant peak in the left SFG medial part close to the HC-vs-CHR-PLB peak (−10, 22, 58, *k* = 18 voxels, *F* = 27.56, *p* = 0.006). Mean *b*-values again confirmed increased activation in PLB relative to HC, with CBD to be intermediate.

### Relationship between behavioural performance and imaging

Within the SN, there was a negative correlation with activity in the left insula/PO in HC (*r* = −0.503, p < 0.001, CI = −0.737 to 0.270; Fig. 2), which was absent in CHR. In CHR-PLB, there was a negative correlation between the *b*-values and mean RT difference between salience and neutral conditions (*r* = −0.308, *p* = 0.028, CI = −0.581 to −0.034; Fig. 2), which was absent in CHR-CBD. Please see Supplementary Analysis for whole brain.

### Relationship between psychopathology and imaging

Within the SN, there was a positive correlation (*r* = 0.569, *p* = 0.017, CI = 0.117 to 1.022) between CAARMS positive score in CHR-PLB and left insula/PO activation (Fig. 2).

## Discussion

In this study, we investigated differences in brain function and behaviour between healthy controls and CHR subjects and examined the effect of a single dose of CBD relative to placebo condition in CHR subjects while processing motivationally salient stimuli. We confirmed our primary hypothesis of abnormal activation within the salience network in CHR-PLB compared to HC, which was modulated by a single dose of CBD. Compared to HC, CHR-PLB had increased activation in the left insula/PO and bilateral FO, associated with premature action initiation. CBD appeared to attenuate activation in the proximate left insula/claustrum, associated with an overall slowing of reaction time. We also established a linear relationship in activation of the left insula/PO and left FO between CHR-PLB, CHR-CBD and HC, with activation intermediate in CHR-CBD. However, we found no differences in activation in the hippocampus-midbrain-striatum between either HC and CHR-PLB or CHR-PLB and CHR-CBD.

Shorter RT during salient compared to neutral stimuli across all groups is consistent with previous literature^43^ and indicates that RT acceleration during the MIDT may be an index of salience perception. In HC, left insula/PO activity negatively correlated with RT during salient stimuli. This may indicate that insular activation is a proxy measure of salience perception and is consistent with the idea that the insula detects salient stimuli to guide behaviour^18^. Such a relationship was absent in the CHR-PLB group. In contrast, activation at this site in CHR-PLB negatively correlated with the RT acceleration during salient compared to neutral stimuli (as indexed by mean RT difference between neutral and salience conditions), indicating that the higher the insular activation, the slower was the acceleration. This may imply that greater insular activation in CHR-PLB relative to HC was associated impaired discrimination of salience in CHR patients and could be a marker of aberrant motivational salience processing. Furthermore, activation at this site positively correlated with CAARMS positive symptoms in CHR-PLB, directly linking aberrant motivational salience processing with psychopathology.

The left insula/PO site of abnormal activation is posteriorly situated and overlaps with the primary site of somatosensory interoceptive input, relaying information to the anterior insula for higher order processing^19^ and switching between the default and central executive networks^44,45^. Left insula function has been implicated in both the generation of psychotic symptoms^28,29,46^ and in antipsychotic treatment^28,47^. Our results extend previous literature by showing that increased activation within the SN was associated with both aberrant processing of motivationally salient stimuli and psychotic symptoms in patients in the very early stages of psychosis. A single dose of CBD attenuated activation in this region, such that it was intermediate between CHR-PLB and HC. However, CBD did not have any effect on striatal or MTL function in the present study, unlike our previous report^36^. This may reflect the different cognitive activation tasks used in the two studies, as in our previous study, we employed a verbal learning task. Here, we did not identify altered striatal or MTL function in CHR-PLB when compared to HC, and a lack of CBD effect may be a consequence. It has been suggested that while the striatum is involved in attribution of motivational salience to stimuli^1^, the insula may be involved with ‘proximal salience’, thought to involve the evaluation of stimuli^30^. Previous studies in CHR patients did not detect any evidence of altered striatal activity^15,42^, consistent with absence of altered striatal activation during the anticipation of motivationally salient stimuli in CHR-PLB relative to HC here. Whether the lack of an effect of diagnosis (CHR-PLB vs HC contrast) or treatment (CBD) on striatal function in the present study reflects a specific dysfunction in ‘proximal salience’, as opposed to motivational salience, and a specific CBD effect on the former, remains to be tested.

The precise molecular mechanism of action of CBD remains unclear. There is evidence that CBD may be a negative allosteric modulator at the CB1 receptor^48^. As CB1 is a presynaptic G-protein coupled inhibitory receptor, CBD could promote neurotransmitter release by inhibiting presynaptic agonism induced by retrograde endocannabinoid messengers. CBD may also enhance endocannabinoid tone by inhibiting breakdown of the CB1 agonist anandamide by fatty acid amide hydrolase^49^. In light of evidence of CB1 receptor alteration in the insula in schizophrenia^50,51^, any antipsychotic effect of CBD may also be through modulation of endocannabinoid dysfunction within the insula.

## Limitations

The present results should be considered in light of certain limitations. Using a within-subject, repeated measures design would have been ideal instead of the cross-sectional design that we have employed, as that would have allowed us to directly test whether CBD normalised altered insular function in CHR patients. Logistical complexities of carrying out such a study influenced our design choice. It is also worth noting that the CHR and HC groups differed in terms of years in education, current cannabis and other drug use that may have influenced brain activation differences between the two groups.

## Conclusion

In summary, results presented here suggest that altered function of the insular cortex, a core component of the salience network, may underlie aberrant salience processing and psychotic symptoms in patients at clinical high-risk of psychosis and that a single dose of CBD may attenuate some of this dysfunction. Future studies need to investigate whether such effects may underlie the antipsychotic effects of CBD observed following a period of treatment.

## Supplementary information

Supplementary Figure 1.

Supplementary Table 1.

Supplementary.

Supplementary Figure 2.

Supplementary Analysis.

## References

[1] Kapur S (2003). Psychosis as a state of aberrant salience: a framework linking biology, phenomenology, and pharmacology in schizophrenia. Am. J. Psychiatry.

[2] Howes OD (2012). The nature of dopamine dysfunction in schizophrenia and what this means for treatment. Arch. Gen. Psychiatry.

[3] Egerton A (2013). Presynaptic striatal dopamine dysfunction in people at ultra-high risk for psychosis: findings in a second cohort. Biol. Psychiatry.

[4] Howes OD (2011). Dopamine synthesis capacity before onset of psychosis: a prospective [18F]-DOPA PET imaging study. Am. J. Psychiatry.

[5] Winton-Brown TT, Fusar-Poli P, Ungless MA, Howes OD (2014). Dopaminergic basis of salience dysregulation in psychosis. Trends Neurosci..

[6] Modinos G, Allen P, Grace AA, McGuire P (2015). Translating the MAM model of psychosis to humans. Trends Neurosci..

[7] Allen P (2015). Resting hyperperfusion of the hippocampus, midbrain, and basal ganglia in people at high risk for psychosis. Am. J. Psychiatry.

[8] Schobel Scott A (2013). Imaging patients with psychosis and a mouse model establishes a spreading pattern of hippocampal dysfunction and implicates glutamate as a driver. Neuron..

[9] Schultz W (2016). Dopamine reward prediction-error signalling: a two-component response. Nat. Rev. Neurosci..

[10] Strauss GP, Waltz JA, Gold JM (2014). A review of reward processing and motivational impairment in schizophrenia. Schizophr. Bull..

[11] Schmidt A (2017). Longitudinal alterations in motivational salience processing in ultra-high-risk subjects for psychosis. Psychol. Med..

[12] Smieskova R (2015). Modulation of motivational salience processing during the early stages of psychosis. Schizophr. Res..

[13] Roiser JP, Howes OD, Chaddock CA, Joyce EM, McGuire P (2012). Neural and behavioral correlates of aberrant salience in individuals at risk for psychosis. Schizophr. Bull..

[14] Winton-Brown T (2017). Altered activation and connectivity in a hippocampal-basal ganglia-midbrain circuit during salience processing in subjects at ultra high risk for psychosis. Transl. Psychiatry.

[15] Wotruba D (2014). Symptom dimensions are associated with reward processing in unmedicated persons at risk for psychosis. Front Behav. Neurosci..

[16] Radua J (2015). Ventral striatal activation during reward processing in psychosis: a neurofunctional meta-analysis. JAMA psychiatry.

[17] Nielsen MØ (2012). Alterations of the brain reward system in antipsychotic naïve schizophrenia patients. Biol. Psychiatry.

[18] Seeley WW (2007). Dissociable intrinsic connectivity networks for salience processing and executive control. J. Neurosci..

[19] Uddin LQ (2015). Salience processing and insular cortical function and dysfunction. Nat. Rev. Neurosci..

[20] Baiano M (2007). Anterior cingulate volumes in schizophrenia: a systematic review and a meta-analysis of MRI studies. Schizophr. Res..

[21] Shepherd AM, Matheson SL, Laurens KR, Carr VJ, Green MJ (2012). Systematic meta-analysis of insula volume in schizophrenia. Biol. Psychiatry.

[22] O’Neill, A., Mechelli, A. & Bhattacharyya, S. Dysconnectivity of large-scale functional networks in early psychosis: a meta-analysis. *Schizophr. Bull.* **45**, 579–590 (2018) 10.1093/schbul/sby094 PMC6483589 29982729

[23] Fornito A (2008). Anatomic abnormalities of the anterior cingulate cortex before psychosis onset: an MRI study of ultra-high-risk individuals. Biol. Psychiatry.

[24] Takahashi T (2009). Insular cortex gray matter changes in individuals at ultra-high-risk of developing psychosis. Schizophr. Res..

[25] Wang C (2016). Disrupted salience network functional connectivity and white-matter microstructure in persons at risk for psychosis: findings from the LYRIKS study. Psychol. Med..

[26] Wotruba D (2014). Aberrant coupling within and across the default mode, task-positive, and salience network in subjects at risk for psychosis. Schizophr. Bull..

[27] Takahashi T (2009). Follow-up MRI study of the insular cortex in first-episode psychosis and chronic schizophrenia. Schizophr. Res..

[28] Walter A (2016). Altered insular function during aberrant salience processing in relation to the severity of psychotic symptoms. Front Psychiatry.

[29] Thusius, N., Romanowicz, M., Mlynek, K. & Sola, C. Prolonged psychosis associated with left insular stroke: talking to God in the walls. *Psychosomatics**.* **59**, 618–621 (2018) 10.1016/j.psym.2018.03.006 29751938

[30] Palaniyappan, L. & Liddle, P. F. Does the salience network play a cardinal role in psychosis? An emerging hypothesis of insular dysfunction. *J. Psychiatry Neurosci*. **37**, 17–27 (2012) 10.1503/jpn.100176 PMC3244495 21693094

[31] Bhattacharyya S (2010). Opposite effects of delta-9-tetrahydrocannabinol and cannabidiol on human brain function and psychopathology. Neuropsychopharmacology.

[32] Englund A (2013). Cannabidiol inhibits THC-elicited paranoid symptoms and hippocampal-dependent memory impairment. J. Psychopharmacol..

[33] Leweke FM (2012). Cannabidiol enhances anandamide signaling and alleviates psychotic symptoms of schizophrenia. Transl. Psychiatry.

[34] McGuire P (2018). Cannabidiol (CBD) as an adjunctive therapy in schizophrenia: a multicenter randomized controlled trial. Am. J. Psychiatry.

[35] Boggs DL (2018). The effects of cannabidiol (CBD) on cognition and symptoms in outpatients with chronic schizophrenia a randomized placebo controlled trial. Psychopharmacology.

[36] Bhattacharyya S (2018). Effect of cannabidiol on medial temporal, midbrain, and striatal dysfunction in people at clinical high risk of psychosis: a randomized clinical trial. JAMA Psychiatry.

[37] Yung AR (2005). Mapping the onset of psychosis: the comprehensive assessment of at-risk mental states. Aust. N. Z. J. Psychiatry.

[38] Knutson B, Westdorp A, Kaiser E, Hommer D (2000). FMRI visualization of brain activity during a monetary incentive delay task. Neuroimage.

[39] Schott BH (2008). Mesolimbic functional magnetic resonance imaging activations during reward anticipation correlate with reward-related ventral striatal dopamine release. J. Neurosci..

[40] Wilson Robin Paul, Colizzi Marco, Bossong Matthijs Geert, Allen Paul, Kempton Matthew, Bhattacharyya Sagnik (2018). The Neural Substrate of Reward Anticipation in Health: A Meta-Analysis of fMRI Findings in the Monetary Incentive Delay Task. Neuropsychology Review.

[41] Nielsen MO (2012). Improvement of brain reward abnormalities by antipsychotic monotherapy in schizophrenia. Arch. Gen. Psychiatry.

[42] Juckel G (2012). Ventral striatal activation during reward processing in subjects with ultra-high risk for schizophrenia. Neuropsychobiology.

[43] Mir P (2011). Motivation and movement: the effect of monetary incentive on performance speed. Exp. Brain Res..

[44] Sridharan D, Levitin DJ, Menon V (2008). A critical role for the right fronto-insular cortex in switching between central-executive and default-mode networks. Proc. Natl Acad. Sci. USA.

[45] Menon V, Uddin LQ (2010). Saliency, switching, attention and control: a network model of insula function. Brain Struct. Funct..

[46] Raij TT, Mantyla T, Mantere O, Kieseppa T, Suvisaari J (2016). Cortical salience network activation precedes the development of delusion severity. Psychol. Med..

[47] Radua J (2012). Multimodal meta-analysis of structural and functional brain changes in first episode psychosis and the effects of antipsychotic medication. Neurosci. Biobehav. Rev..

[48] Laprairie R, Bagher A, Kelly M, Denovan‐Wright E (2015). Cannabidiol is a negative allosteric modulator of the cannabinoid CB1 receptor. Br. J. Pharmacol..

[49] Bisogno T (2001). Molecular targets for cannabidiol and its synthetic analogues: effect on vanilloid VR1 receptors and on the cellular uptake and enzymatic hydrolysis of anandamide. Br. J. Pharmacol..

[50] Ranganathan M (2016). Reduced brain cannabinoid receptor availability in schizophrenia. Biol. Psychiatry.

[51] Ceccarini J (2013). Increased ventral striatal CB1 receptor binding is related to negative symptoms in drug-free patients with schizophrenia. NeuroImage.

