# Supplementary figures and images for: Cannabidiol attenuates insular dysfunction during motivational salience processing in subjects at clinical high risk for psychosis
Source: Transl Psychiatry. 2019 Aug 22;9:203. doi: 10.1038/s41398-019-0534-2 (PMC6706374; doi:10.1038/s41398-019-0534-2)

Figure 1. Example of MIDT visual cue sequence


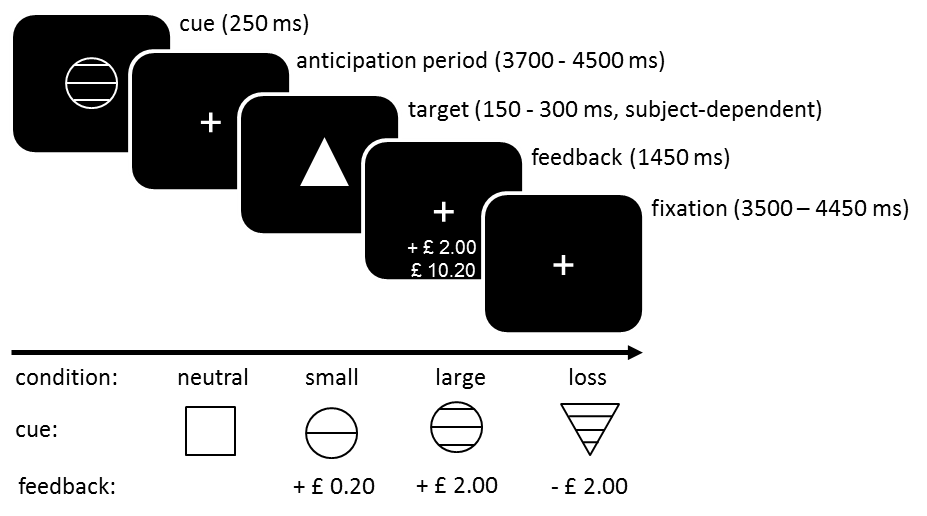

Supplement: Supplementary file 1 — Supplementary Figure 1. [file 41398_2019_534_MOESM1_ESM.docx]
