# Supplementary material for: Cannabidiol attenuates insular dysfunction during motivational salience processing in subjects at clinical high risk for psychosis
Source: Transl Psychiatry. 2019 Aug 22;9:203. doi: 10.1038/s41398-019-0534-2 (PMC6706374; doi:10.1038/s41398-019-0534-2)
Supplement: Supplementary file 2 — Supplementary Table 1. [file 41398_2019_534_MOESM2_ESM.docx]

Table 1. Brain regions activated by the monetary incentive delay task in healthy controls

| Region | Peak coordinate (MNI) | | | Cluster size | p(FWE-corr) |
| --- | --- | --- | --- | --- | --- |
|  | x | y | z |  |  |
| **Core salience network ROI analysis**  Right parietal operculum  Left insula  Right insula/inferior frontal gyrus triangular part | 34  32  -30  32 | -40  -32  -38  22 | 18  14  18  12 | 47  19  11 | 0.001  0.003  0.002  0.018 |
| **Hippocampus-midbrain-striatum ROI analysis**  Right amygdala | 20  20  -14 | -8  -12  -26 | -6  4  -18 | 3578 | <0.001 |
| **Wholebrain analysis**  Left precentral gyrus  Right amygdala  Left occipital gyrus (nearest GM)  Right anterior cingulate  Left occipital gyrus  Left paracentral lobule  Left cerebellum posterior lobe  Right cerebellum posterior lobe  Right lateral orbital gyrus  Left paracentral lobule  Left superior parietal lobule  Right inferior frontal gyrus orbital part  Right superior parietal lobule  Right occipital gyrus  Right cerebellum posterior lobe | -54  -20  14  18  20  -18  -34  -24  16  14  -28  -16  -16  32  22  36  30  -8  -18  28  28  16  8  20 | -2  -2  4  -8  -14  -18  -50  -70  -82  20  -94  -24  -44  -66  -62  -76  34  -44  -54  24  36  -70  2  -46 | 44  56  56  -8  4  6  6  6  8  32  8  40  -44  -28  -26  -26  0  58  58  10  12  56  42  -44 | 6853  6767  909  98  70  28  33  95  22  33  13  21  20  13  11 | <0.001  <0.001  <0.001  <0.001  <0.001  <0.001  <0.001  <0.001  <0.001  <0.001  0.002  0.006  0.007  0.013  0.019  0.037  0.014  0.015  0.015  0.015  0.033  0.024  0.032  0.033 |

Table 1. Brain regions activated by the monetary incentive delay task in healthy controls for motivational salience (salience>neutral) in salience network region of interest (ROI), hippocampus-midbrain-striatum ROI and whole-brain. 1-sample t-test, FWE-corrected p<0.05, k>10 voxels
