# Supplementary material for: Cannabidiol attenuates insular dysfunction during motivational salience processing in subjects at clinical high risk for psychosis
Source: Transl Psychiatry. 2019 Aug 22;9:203. doi: 10.1038/s41398-019-0534-2 (PMC6706374; doi:10.1038/s41398-019-0534-2)
Supplement: Supplementary file 3 — Supplementary. [file 41398_2019_534_MOESM3_ESM.docx]

Table 2. Wholebrain analysis for salience-vs-neutral contrast

| Region | Peak coordinate (MNI) | | | Cluster size | p |
| --- | --- | --- | --- | --- | --- |
|  | x | y | z |  |  |
| **Pairwise comparison**  **CHR-PLB>HC** | | | | | |
| Left superior frontal gyrus medial part | -8 | 24 | 58 | 141 | <0.001 |
| Left inferior frontal gyrus opercular part  (Left frontal operculum) | -54 | 20 | 20 | 65 | 0.002 |
|  | -44 | 16 | 12 |  | 0.004 |
| Left superior temporal gyrus/supramarginal gyrus | -54 | -54 | 30 | 13 | 0.009 |
| **Pairwise comparison**  **CHR-PLB>CHR-CBD** | | | | | |
| Right superior frontal gyrus lateral part | 20 | -4 | 48 | 3 | 0.025 |
| **Pairwise comparison**  **CHR-CBD>CHR-PLB** | | | | | |
| Right cerebellum posterior lobe | 6 | -82 | -38 | 6 | 0.022 |
| **3-way ANOVA**  **CHR-PLB>CHR-CBD>HC** | | | | | |
| Left superior frontal gyrus medial part | -10 | 22 | 58 | 18 | 0.006 |

Table 2. Wholebrain analysis for salience-vs-neutral contrast. Family wise error-corrected p<0.05, k≥3voxels. Abbreviations: HC=healthy control group, CHR-CBD=clinical-high risk cannabidiol group, CHR-PLB=clinical-high risk placebo group.
