# Supplementary material for: Cannabidiol attenuates insular dysfunction during motivational salience processing in subjects at clinical high risk for psychosis
Source: Transl Psychiatry. 2019 Aug 22;9:203. doi: 10.1038/s41398-019-0534-2 (PMC6706374; doi:10.1038/s41398-019-0534-2)
Supplement: Supplementary file 4 — Supplementary Figure 2. [file 41398_2019_534_MOESM4_ESM.docx]

Figure 2. Whole-brain analysis of salience>neutral contrast


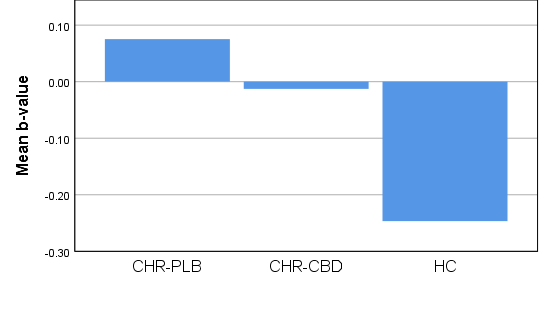


E

F


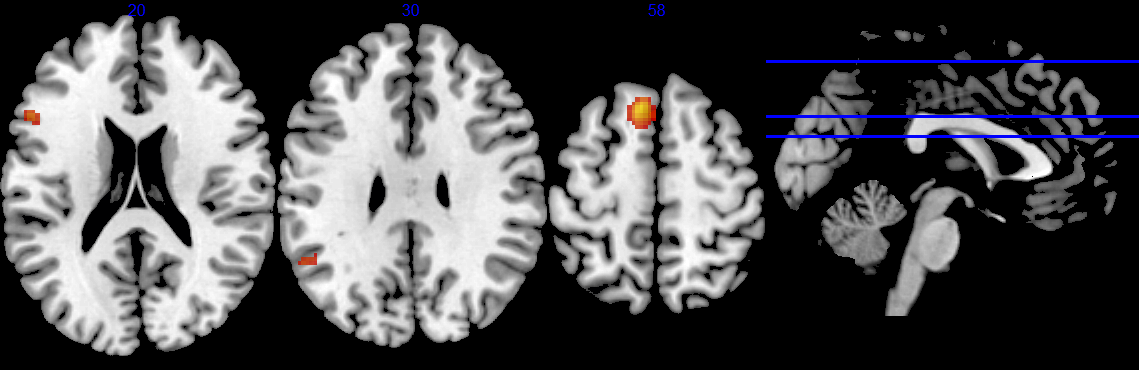

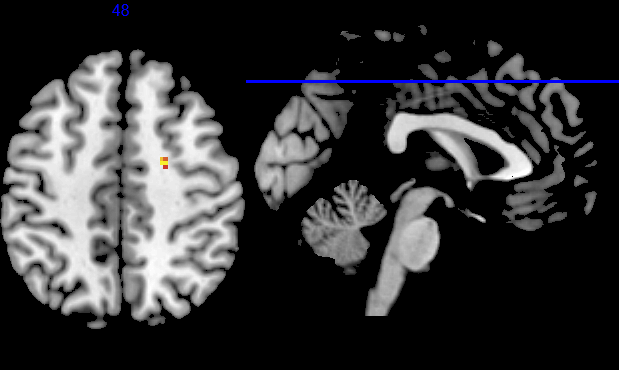

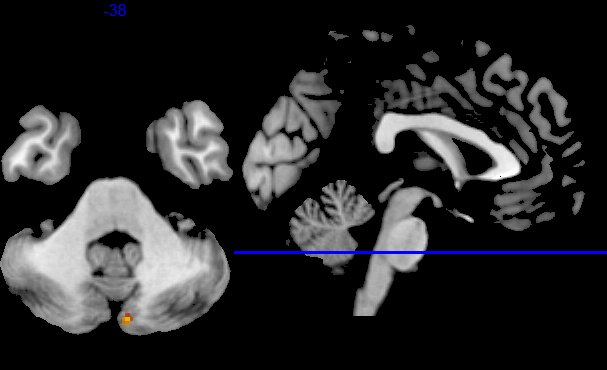

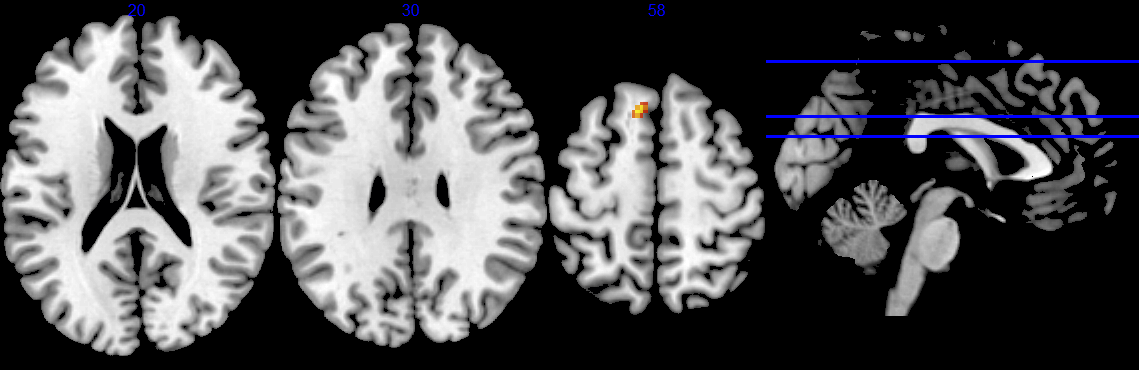

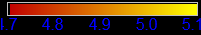

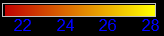

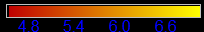


A

B

D

C


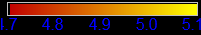

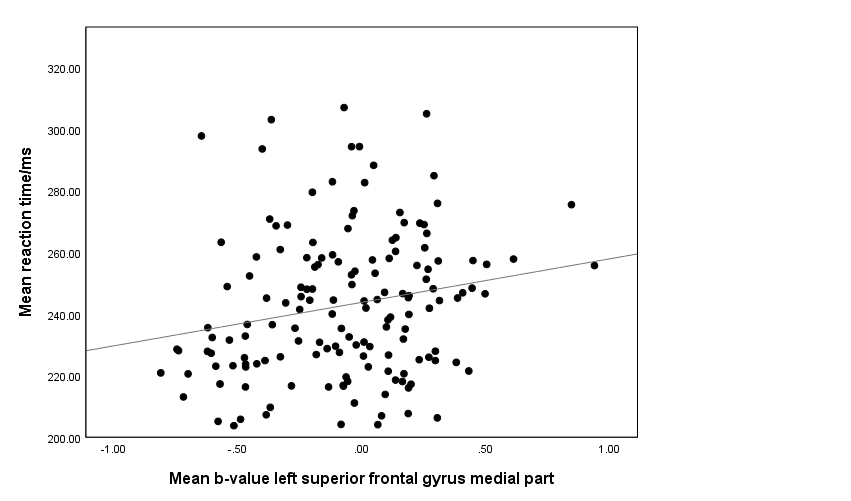


Figure 2. Whole-brain analysis of salience>neutral contrast (small-volume corrected, p<0.05 FWE-corrected at voxel level, k≥3 voxels). (A) Pairwise comparison CHR-PLB>HC with clusters in left superior frontal gyrus medial part, left inferior frontal gyrus opercular part extending to left frontal operculum and left superior temporal gyrus. (B) Pairwise comparison CHR-PLB>CHR-CBD with cluster in right superior frontal gyrus lateral part. (C) Pairwise comparison CHR-CBD>CHR-PLB with cluster in right cerebellum posterior lobe. (D) 3-way ANOVA CHR-PLB>CHR-CBD>HC with cluster in left superior frontal gyrus medial part. (E) Mean b-value parameter estimates extracted from ANOVA-derived cluster in the left superior frontal gyrus medial part for each group (CHR-PLB, CHR-CBD, and HC) showing increased activation in CHR-PLB relative to HC with CHR-CBD intermediate. (F) Positive correlation between mean b-value from ANOVA-derived cluster of left superior frontal gyrus medial part and mean reaction time for salience condition in CHR-CBD.
