# Supplementary material for: Cannabidiol attenuates insular dysfunction during motivational salience processing in subjects at clinical high risk for psychosis
Source: Transl Psychiatry. 2019 Aug 22;9:203. doi: 10.1038/s41398-019-0534-2 (PMC6706374; doi:10.1038/s41398-019-0534-2)
Supplement: Supplementary file 5 — Supplementary Analysis. [file 41398_2019_534_MOESM5_ESM.docx]

Supplementary Analysis

In the exploratory whole-brain analysis, we report increased activation in the left SFGM, left IFGOP and left STG in HC-vs-CHR-PLB. In CHR-PLB-vs-CHR-CBD there was relative deactivation of the right SFGL and increased activation in the right cerebellum. A linear relationship was confirmed by ANOVA in the left SFGM, with CBD attenuating activation. This in turn correlated with reaction time during salience (r=0.377, p=0.011, CI=0.093-0.662; Supplementary Table 2, Supplementary Figure 2) absent in CHR-PLB and HC. CBD was associated with an overall slowing of motor response, suggesting a role modulating premature action. The pre-SMA region is thought to be critical to behavioural response selection and inhibition in measures of response inhibition (1).

1. Simmonds DJ, Pekar JJ, Mostofsky SH. Meta-analysis of Go/No-go tasks demonstrating that fMRI activation associated with response inhibition is task-dependent. Neuropsychologia. 2008;46(1):224-32.
